# A systematic overview of dental methods for age assessment in living individuals: from traditional to artificial intelligence-based approaches

**DOI:** 10.1007/s00414-023-02960-z

**Published:** 2023-04-14

**Authors:** Nicolás Vila-Blanco, Paulina Varas-Quintana, Inmaculada Tomás, María J. Carreira

**Affiliations:** 1 Centro Singular de Investigación en Tecnoloxías Intelixentes (CiTIUS), Universidade de Santiago de Compostela, Santiago de Compostela, Spain; 2 Departamento de Electrónica e Computación, Escola Técnica Superior de Enxeñaría, Universidade de Santiago de Compostela, Santiago de Compostela, Spain; 3 Instituto de Investigación Sanitaria de Santiago de Compostela (IDIS), Santiago de Compostela, Spain; 4 Oral Sciences Research Group, Special Needs Unit, Department of Surgery and Medical Surgical Specialities, School of Medicine and Dentistry, Universidade de Santiago de Compostela, Santiago de Compostela, Spain

**Keywords:** Dental radiology, Chronological age estimation, Forensic dentistry, Deep learning

## Abstract

Dental radiographies have been used for many decades for estimating the chronological age, with a view to forensic identification, migration flow control, or assessment of dental development, among others. This study aims to analyse the current application of chronological age estimation methods from dental X-ray images in the last 6 years, involving a search for works in the Scopus and PubMed databases. Exclusion criteria were applied to discard off-topic studies and experiments which are not compliant with a minimum quality standard. The studies were grouped according to the applied methodology, the estimation target, and the age cohort used to evaluate the estimation performance. A set of performance metrics was used to ensure good comparability between the different proposed methodologies. A total of 613 unique studies were retrieved, of which 286 were selected according to the inclusion criteria. Notable tendencies to overestimation and underestimation were observed in some manual approaches for numeric age estimation, being especially notable in the case of Demirjian (overestimation) and Cameriere (underestimation). On the other hand, the automatic approaches based on deep learning techniques are scarcer, with only 17 studies published in this regard, but they showed a more balanced behaviour, with no tendency to overestimation or underestimation. From the analysis of the results, it can be concluded that traditional methods have been evaluated in a wide variety of population samples, ensuring good applicability in different ethnicities. On the other hand, fully automated methods were a turning point in terms of performance, cost, and adaptability to new populations.

## Introduction

Chronological age is, together with biological sex and ethnicity, the most important human feature to be considered in anthropological and forensic studies [1]. Besides, chronological age estimation is used daily in legal procedures where the birthdate of the involved subjects can not be verified due to either the absence of birth certification or the suspicion of false documentation. This applies to migration controls or trials involving undocumented people since the attainment of legal age has many implications according to the laws of most countries. It is also important in the adoption processes of undocumented children. In all these cases, an expert performs a somatic maturity examination.

The development status of bones has been used successfully to estimate chronological age. In this regard, many skeletal parts have been used, such as pubic symphysis, auricular surface, or sternal ribs [2]. Also, it is worth noting that there is not a single method based on bone development that outperforms others systematically, as the performance of each one depends on numerous factors. For instance, there are specific age estimation methods developed for subadults and others that work better in adults [3].

One of the most widely used body part in the field of age estimation is the teeth, mainly because dental mineralisation has been reported to be less affected by external factors (e.g. genetics or environment) than bone mineralisation [4]. In this regard, dental imaging techniques represented a step forward because they allowed clinicians to assess bone development with less invasive and faster procedures, and thus enabled them to perform chronological age estimation.

The estimation of age from dental radiographic records is based on the evaluation of some characteristics such as the formation of jaw bones; the appearance of tooth germs, the degree of crown completion and its eruption, the degree of resorption of deciduous teeth; the measurement of open apices in teeth; the volume of the pulp chamber and root canals; the formation of physiological secondary dentin; the toot-to-pulp ratio; or the development and topography of the third molar [5].

It is worth noting that the panoramic X-rays (ortopantomographies or OPGs) provide the least invasive radiologic technique to estimate age, as it only requires a single image to capture the whole dentition. Besides, other bone structures can be seen, such as the mandible, the nasal fossa, or the vertebrae, which are also helpful for further examinations. In the following, a review of the main methods to estimate the age of dental radiographs has been carried out.

## Material and methods

**Fig. 1:**
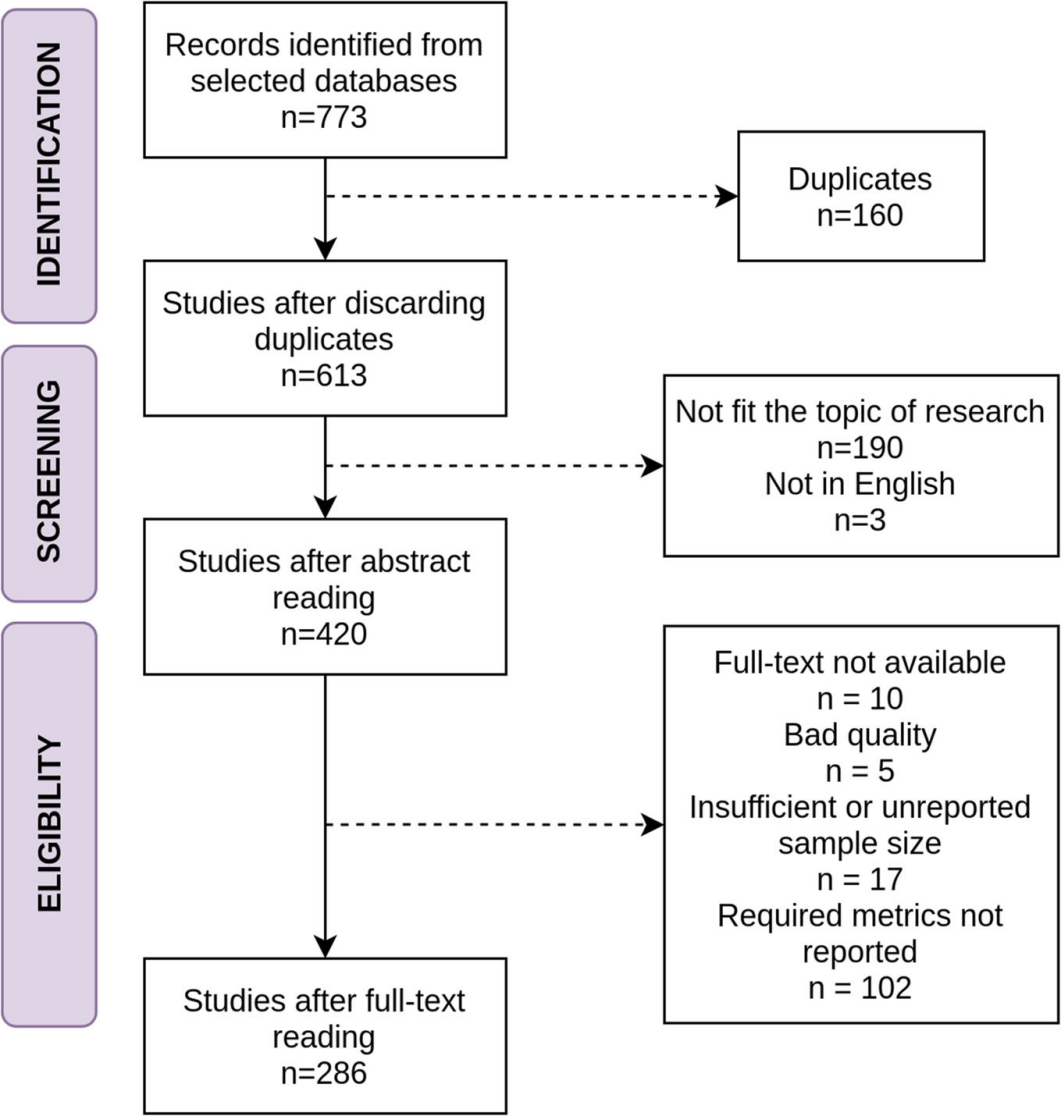
Flow diagram of the study selection process

For the review purpose, a conducting protocol approved by an expert reviewer and compliant with the PRISMA guidelines for systematic reviews [6] has been established. Scopus and PubMed databases have been used to retrieve a collection of full-text studies on age estimation from dental radiographies published in the last 6 years (from 2016 to 2022). This specific period was chosen for two main reasons. First, the number of published studies is sufficiently high to report significant conclusions. Second, automatic methodologies in the field of dental age estimation have been mainly used in this period, and not before, and therefore including earlier years would have diluted their relevance in this review. Then, a study selection process has been carried out, as seen in Fig. 1.

The query used in Scopus was:

- TITLE-ABS-KEY ((“age estimation” OR “age assessment” OR “age regression” OR “age determination” ) AND (dental OR tooth OR teeth OR mandib* OR incisor OR canine OR premolar OR molar ) AND (x-ray OR radiolog* OR radiograph* OR opg OR orthopantomograph* OR panoramic OR ct OR cbct OR mri ) ) AND (LIMIT-TO (DOCTYPE , “ar” ) OR LIMIT-TO (DOCTYPE , “re” ) OR LIMIT-TO (DOCTYPE , “cp” ) ) AND (LIMIT-TO (PUBYEAR , 2022 ) OR LIMIT-TO (PUBYEAR , 2021 ) OR LIMIT-TO (PUBYEAR , 2020 ) OR LIMIT-TO (PUBYEAR , 2019 ) OR LIMIT-TO (PUBYEAR , 2018 ) OR LIMIT-TO (PUBYEAR , 2017 ) OR LIMIT-TO (PUBYEAR , 2016 ) ) AND (LIMIT-TO (LANGUAGE , “English” ) ) AND (EXCLUDE (SUBJAREA , “BIOC” ) ) AND (EXCLUDE (SUBJAREA , “PHAR” ) OR EXCLUDE (SUBJAREA , “PHYS” ) OR EXCLUDE (SUBJAREA , “VETE” ) OR EXCLUDE (SUBJAREA , “ARTS” ) OR EXCLUDE (SUBJAREA , “EART” ) OR EXCLUDE (SUBJAREA , “MATE” ) OR EXCLUDE (SUBJAREA , “BUSI” ) OR EXCLUDE (SUBJAREA , “CENG” ) OR EXCLUDE (SUBJAREA , “CHEM” ) OR EXCLUDE (SUBJAREA , “ENER” ) OR EXCLUDE (SUBJAREA , “PSYC” ) )

The query used in PubMed was:

- (age estimation [Title/Abstract] OR age assessment [Title/Abstract] OR age regression [Title/Abstract] OR age determination [Title/Abstract]) AND (dental [Title/Abstract] OR tooth [Title/Abstract] OR teeth [Title/Abstract] OR incisor [Title/Abstract] OR canine [Title/Abstract] OR premolar [Title/Abstract] OR molar [Title/Abstract] OR mandib* [Title/Abstract]) AND (x-ray [Title/Abstract] OR radiograph* [Title/Abstract] OR radiolog* [Title/Abstract] OR opg [Title/Abstract] OR orthopantomograph* [Title/Abstract] OR panoramic [Title/Abstract] OR CT [Title/Abstract] OR MRI [Title/Abstract] OR CBCT [Title/Abstract]) AND 2016[DP]: 2022[DP] AND full text [SB] AND english [LA]

As it can be seen, the query is not strictly the same, as Scopus allowed also for excluding certain unwanted subject areas, such as Veterinary or Arts. As a result, a set of 537 studies were collected from Scopus and 336 from Pubmed on February 24th, 2022, which in the end represented a body of 613 unique works. The abstract of each work was reviewed to discard unwanted studies, according to the following exclusion criteria: (1) studies not aimed at chronological age estimation in humans; (2) non-radiological studies; (3) studies that use non-human samples; (4) studies relying on a sample smaller than 50 subjects or studies that do not report the sample size; (5) studies whose full text is not available.

Regarding the collection of studies aimed at evaluating the age estimation methods proposed in the literature, only those reporting at least one of the following metrics were evaluated. In terms of numerical age estimation studies, a statistic on the residual error (dental age minus chronological age) and the absolute error—mean, median, or standard deviation—, the standard error of the estimates, and/or the coefficient of determination *R*^2^. Methods geared toward age classification were required to report the accuracy, sensitivity, and/or specificity of the classification results. Although dental development is less affected by genetic or environmental factors than other bones, this process is still subject to variations, and so the age estimation methods were usually assessed in different populations and/or ethnic groups all over the world.

To reduce as much as possible the risk of bias in this work when comparing the results obtained by different methods, the collected studies were analysed to detect evidence of malpractice. As a result, five studies were discarded due to the non-compliance with basic aspects such as good wording or a comprehensive description of the experiments, as this could also indicate a problem in the peer review process. It is worth noting that only the most flagrant cases were taken into account to minimise the bias that the observer could introduce in this evaluation process. In the end, a set of 286 studies was selected for further analysis.

## Age estimation methods

### Tooth-based manual methods

**Fig. 2:**
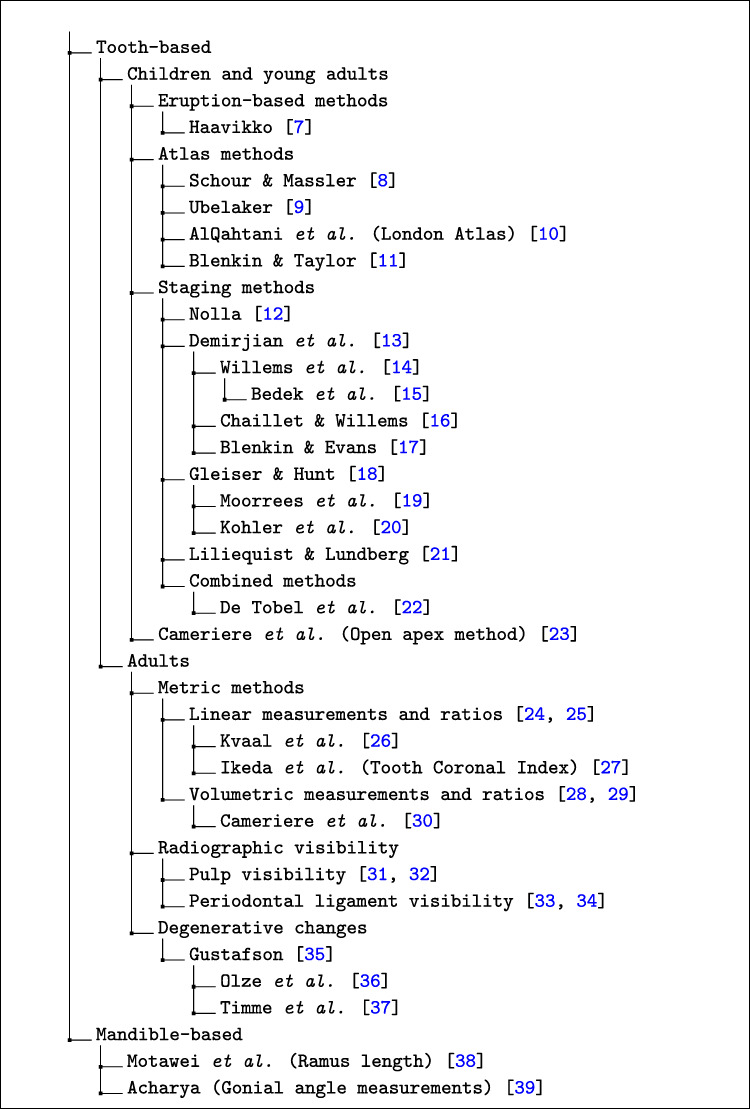
Main manual methods for estimating chronological age from dental X-ray images

The studies retrieved in this work relied on a wide variety of age estimation methodologies. However, as dental formation is highly correlated with chronological age and, therefore, is a key indicator for age estimation, most methods are based on dentition analysis. In this regard, the first approaches were purely manual, that is, they required experts not only to retrieve the correspondent information from the X-ray image but also to translate this information into an age value. These approaches [7–39] are shown in Fig. 2.

#### Children and young adults

Age estimation via dentition analysis has reportedly led to better results when dealing with newborns to subjects aged 22 to 25, that is, during tooth development. This makes studies aimed at estimating the age of children and/or adolescents to be more numerous than those focusing on age estimation in adults. Regarding the former, some methods aim to assess specific development milestones (such as dental eruption) to predict age [7, 40]. Though, they have proven to lead to very limited estimates, as they rely on very quick changes, from which little information can be collected.

Other methods aimed to assess the development of the teeth over a longer period. That is the case for dental Atlases, which are graphic representations of dental development and eruption that provide an easy way to estimate chronological age by comparing the status of the dentition using radiological or osteological techniques to the charts provided in the Atlas [8–11]. Other authors went a step further and developed dental scoring systems (DSS), consisting of dividing the development period of each tooth into a set of developmental stages with associated scores and using those scores to estimate the numerical chronological age. In this regard, the number of stages varied depending on the specific system. For example, Gleiser and Hunt [18] proposed 15 stages, Nolla [12] developed a division into 11 stages, Demirjian et al. [13] reported eight alphabetical stages, and Liliequist and Lundberg [21] proposed the use of seven stages, in a clear attempt to reduce the complexity of the method. Furthermore, some authors developed population-specific scoring tables on top of the Demirjian et al. [14, 16, 17] and Gleiser and Hunt’s systems [19, 20], while others mixed several staging systems to improve the overall estimation performance [22].

Cameriere et al. [23] introduced a different method for estimating age, based on tooth measurements. Specifically, the authors measured the open apices of the seven left permanent mandibular teeth. These measurements, previously normalised by tooth height, were highly and negatively correlated with chronological age. Furthermore, the number of teeth with completely closed root apices was reportedly correlated with age. These findings led the authors to develop a regression formula that depends on the sex of the subject and the normalised measurements of the seven teeth and the number of teeth whose root development is completed.

#### Adults

Although the development of teeth ends once the third molar is completely developed, some authors focused on other age-related changes that are radiologically observable to estimate age in older subjects. In this regard, three different families of methods can be identified. On the one hand, some authors explored the use of specific measurements or ratios between them to perform age estimations. For example, Kvaal et al. [26] proposed to measure dentin apposition indirectly through the assessment of the dental pulp radiopacity. The researchers carried out several linear measurements of both the pulp and the tooth and associated those measurements via linear ratios. Cameriere et al. [30] proposed a similar idea, but they replaced the linear measurements with area assessments. Another similar example is the Tooth Coronal Index (TCI), studied by Ikeda et al. [27], which represents a height ratio between the crown and the pulp cavity at the crown level.

On the other hand, a set of studies focused on the visibility of some structures established staging systems with which that visibility could be assessed. The structures most studied in this regard were the periodontal ligament and the root pulp, with the staging systems proposed in this regard by Olze et al. [31, 33] standing out.

Finally, some authors reported that a series of degenerative changes can be assessed through a staging system and therefore be used to estimate chronological age. In this regard, Gustafson [35] set multiple evaluable criteria, namely secondary dentin formation, periodontal recession, attrition, apical translucency, cementum apposition, and external root resorption. The degenerative stages proposed in the original work, which were intended to be applied to extracted and ground teeth, proved to be applicable to radiographic images as well, as confirmed with the methodologies proposed by other authors, such as Olze et al. [36] and Timme et al. [37].

#### Non-numeric age estimation

Besides age estimation methods developed for obtaining a numeric and continuous output, other authors focused on designing classification methods to estimate the probability that a subject belongs to a specific age group. Most of these studies relied on conventional numerical age estimation methods and adapted them to be used as age group classifiers. This is the case, for example, of the study of Sehrawat and Singh [41], which used the Kvaal et al.’s method [26] to perform a classification into four groups.

However, the majority of these studies are focused on a binary classification with two groups of subjects younger and older than a given threshold, which can be the legal age of maturity or any other specific age with high relevance in legal procedures. In this regard, Mincer et al. [42] relied on the staging system proposed by Demirjian et al. [13] to assess the development of the third molar, with the objective of estimating the probability of being older than a certain age for each stage. De Luca et al. [43] used measurements of the open apices to establish a cut-off value of 0.08 over the normalised Cameriere measurement of the third molar, above which the individual is considered older than the legal age. Other numeric age estimation methods were evaluated in age classification problems, such as Nolla’s [44] and Kohler et al.’s [45].

#### Age estimation on other radiologically observable structures

Although most age estimation methods from dental radiologic records are based on the analysis of the teeth, there are other structures whose characteristics may also be useful for age estimation. In the period covered by this systematic review (from 2016 to 2022), the number of works is very limited and all of them rely on mandibular measurements. Some examples are the approach followed by Motawei et al. [38], who established a relationship between the length of the ramus and chronological age, or the proposal by Acharya [39], in which the gonial angle was used as the main age indicator.

### Automatic methods

Recent advances in image processing have allowed for automating dental age estimation methods to a greater degree and have led to the development of numerous methodologies. In this regard, the authors explored the same objectives as those covered in the traditional methods, as can be seen in Table 1: numeric age estimation [46–55] and age group classification [56–60], with the particular case of age thresholding [47, 61].

**Table 1:** Main automatic methods for estimating age. *OPG*, ortopantomograph; *FA*, fully automatic; *CNN*, convolutional neural network; *NAS*, neural architecture search; *SHN*, stacked hourglass network; *NA*, numeric age; *AGC*, age group classification; *AT*, age thresholding; *M*_*i*_, *i* th molar; *PM*_*i*_, *i* th premolar

Reference	Image	FA	Method	Target	Required teeth
Čular et al. [46]	OPG	$\checkmark $	Active Appearance Model	NA	− Right mandibular *M*_3_
			+ Radial Basis Network		
Štern et al. [47]	MRI	*χ*	Manual ROI crop	NA + AT	No specific tooth required
			+ Regression CNN		
De Back et al. [48]	OPG	$\checkmark $	Bayesian CNN	NA	No specific tooth needed
Vila-Blanco et al. [49]	OPG	$\checkmark $	Two-path CNN	NA	No specific tooth needed
Hou et al. [50]	OPG	$\checkmark $	CNN designed through NAS	NA	No specific tooth needed
Pham et al. [51]	CT	$\checkmark $	Thresholding mandible segmentation	NA	No specific tooth needed
			+ 3D ResNet 34		
Wallraff et al. [52]	OPG	$\checkmark $	ResNet18	NA	No specific tooth needed
Zheng et al. [53]	CBCT	*χ*	Manual ROI crop	NA	− *M*_1_ in all four quadrants
			+ Custom CNN segmentation		
			+ Level set segmentation refinement		
			+ Linear regression		
Vila-Blanco et al. [54]	OPG	$\checkmark $	SHN mandible segmentation	NA	No specific tooth needed
			+ PDM shape characterisation		
			+ Ridge regression		
Milošević et al. [55]	OPG	$\checkmark $	VGG16	NA	No specific tooth needed
			+ Attention mechanism		
De Tobel et al. [56]	OPG	*χ*	Manual ROI crop	AGC	− Left mandibular *M*_3_
			+ Staging CNN		
Merdietio et al. [57]	OPG	$\checkmark $	Segmentation CNN	AGC	− Left mandibular *M*_3_
			+ Staging CNN		
Banar et al. [58]	OPG	$\checkmark $	Localization CNN	AGC	− Left mandibular *M*_3_
			+ Segmentation CNN		
			+ Staging CNN		
Kim et al. [59]	OPG	*χ*	Manual ROI crop	AGC	− *M*_3_ in all four quadrants
			+ Staging CNN		
Kahaki et al. [60]	OPG	$\checkmark $	Global fuzzy segmentation	AGC	− Left mandibular *M*_1_, *M*_2_ and *M*_3_
			+ Intensity projection		
			+ Staging CNN		
Guo et al. [61]	OPG	$\checkmark $	SE-ResNet 101	AT	No specific tooth needed

Among the methods developed for numerical age estimation, some authors focused on subjects with developing dentitions (up to 20 or 25 years old), such as Čular et al. [46], De Back et al. [48], and Wallraff et al. [52], while others were targeted at a wider range of age cohorts, such as Vila-Blanco et al. [49], Zheng et al. [53], Hou et al. [50], and Milošević et al. [55]. It is noticeable that Vila-Blanco et al. [54] also developed an automatic method to estimate age in children and subadults that is not focused on the entire panoramic image or the dental area but the shape of the mandible.

On the other hand, some methods were designed to perform an age group classification, with a variable number of target groups. While De Tobel et al. [56], Merdietio et al. [57], and Banar et al. [58] treated the problem as a ten-stage, Kim et al. [59] and Kahaki et al. [60] reduced the number of stages to five. Moreover, Guo et al. [61] focused on the binary classification problem of legal age detection, establishing the thresholds of 14, 16, and 18 years.

Regarding the applied methodologies, one of the first attempts to rely on image processing techniques was made by Čular et al. [46], where the authors proposed the use of an Active Appearance Model to localise the third molar and parameterise its shape and texture. In a second step, these parameters are introduced into a neural network to estimate the chronological age. As both steps do not need human intervention, the method works automatically.

As in most of the topics involving image processing, deep neural networks (DNNs) helped not only to automatise some tasks but also to improve their performance. Regarding age estimation, De Tobel et al. [56] developed a staging system for the third molar based on modified Demirjian stages and used a DNN to classify the third molar image crops into one of those stages. This method only required a minimum intervention of the expert to crop the region of interest to frame the third molar area. This approach was updated by Merdietio et al. [57] by replacing the manual crop step with a DenseNet network, which allows estimation to run automatically. Banar et al. [58] developed a similar method, with a slightly more complex third molar segmentation, in which the tooth is first localised and then segmented.

Kim et al. [59] followed a similar approach to that of De Tobel et al. [56]. The authors also developed a two-step approach which firstly requires a manual crop of the third molar, although in this case the four third molars are required. In the second step, each of the four teeth is classified into different age groups, and the classifications are merged through a majority voting system. The authors established two different age group divisions: the first grouped subjects younger than 20, subjects aged 20 to 49, and those over 50; the second split the middle group into three subgroups, namely subjects aged 20 to 29, subjects aged 30 to 39, and those aged 40 to 49.

Although deep learning methods had already been introduced in the studies mentioned above, De Back et al. [48] proposed the use of a DNN, specifically a Bayesian Convolutional Neural Network, as the only step to estimate chronological age. Therefore, the expert does not need to specify which features of the image should be taken into account, as the network focuses automatically on those regions which contributes the most to the age estimation. Furthermore, the age estimation process can proceed even if several teeth are missing.

Vila-Blanco et al. [49], following the clinical finding that dental development is different in boys and girls, developed a method to automatically integrate sexual information into the age estimation process. Thus, they proposed the use of two identical CNNs, one for age estimation and the other for sex classification, so that the sex CNN learns sexual dimorphic features and propagates them to the age CNN at intermediate points to improve the age estimation performance.

Similarly to that proposed by De Back et al. [48] and Vila-Blanco et al. [49], Hou et al. [50], Wallraff et al. [52], Milošević et al. [55], and Guo et al. [61] also developed one-stage methods that do not require the presence of specific teeth to work.

## Evaluation studies

**Fig. 3:**
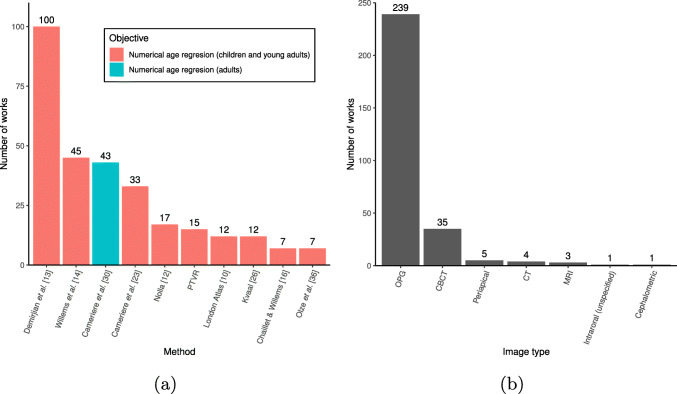
**a** Number of studies applying the ten most assessed methods for age estimation in dental radiographic images in the period evaluated in this review (2016 to 2022), where PTVR represents the works that used pulp-to-tooth volumetric ratios; **b** Number of studies relying on the different types of radiological dental images used in the period evaluated in this review (2016 to 2022)

The studies retrieved in this work were categorised according to the age estimation methods they relied on. As it can be seen in Fig. 3a, where the ten most used methods are represented, Demirjian et al.’s approach [13] has been applied in more than a third of the studies (100 out of 286), with some methods derived for it also in the first positions—Willems et al.’s [14] and Chaillet and Willems’ [16] were applied in 45 and 7 studies, respectively. The first method not aimed originally at estimating the numerical age is the approach proposed by Cameriere et al. [62]. This method, focused on the classification of subjects younger or older than legal age, was used in 43 studies. On the other hand, it is noticeable that 239 studies relied on OPGs to conduct the experiments, representing 84% of all the retrieved works (Fig. 3b). The rest of the studies used CT-based techniques (such as CBCT or conventional CT) and, to a lesser extent, intraoral images, MRI, or the cephalometric view.

Regarding the performance of the age estimation methods, a maximum of one study was evaluated for each population and each method, specifically that evaluated in the largest sample due to the greater significance of the reported results. This ensured a good representation of different ethnicities while avoiding overcrowded result tables. Following the same order as in the previous section, the approach based on tooth eruption assessment proposed by Haavikko [7] was evaluated in a wide range of populations since its development, but it clearly lost popularity in comparison to other methods. As shown in Table 2, only four studies that met the inclusion criteria have been analysed, all focused on subjects younger than 16. In terms of performance, these studies reported systematic underestimations given by residual errors (difference between estimated age and real age) with means ranging from − 0.22 to − 1.35 years and standard deviations around one year. The mean absolute errors yielded mean values of 0.33 to 1.45 years.

**Table 2:** Evaluation of the Haavikko’s method [7]

Evaluation work	Population	*n*	Age	E (DA-CA)			AE (|DA-CA|)			SE	*R*^2^
				*μ*	*σ*	med.	*μ*	*σ*	med.		
Hedge et al. [81]	Indian	1200	5–15	− 0.22	0.82	–	0.71	–	–	–	–
Kumaresan et al. [82]	Malaysian	426	5–15	− 1.31	1.10	–	1.45	–	–	–	–
Benedicto et al. [83]	Brazilian	1059	8–16	− 1.35	1.05	–	1.42	–	1.29	–	–
Sezer & Çarıkçıoğlu [84]	Turkish	980	6–15	− 0.49	0.85	–	0.33	0.26	–	–	–

The atlas-based methods listed in Fig. 2 were also applied in the last few years, although only the London Atlas proposed by AlQahtani et al. [10] was tested in more than one population sample. As shown in Table 3, the work by Baylis and Bassed [63], which compared the three Atlas-based methods in a New Zealander population, reported a slight underestimation with the Schour and Massler Atlas [8] (− 0.03 to − 0.39 years of mean error), a slight overestimation with the Blenkin and Taylor method [11] (+ 0.07 to + 0.34 years), and a noticeable overestimation with the London Atlas [10] (+ 0.40 to + 0.74 years). The latter resulted in a systematic overestimation in the rest of the populations in which it was applied. In terms of absolute error, the mean values ranged from 0.43 to 1.43 years, while the standard deviations ranged between 0.36 and 1.05.

**Table 3:** Evaluation of Atlas-based methods to estimate age in subadults. When two values are given, they correspond to the metrics obtained in males/females

Evaluation work	Population	*n*	Age	Atlas	E (DA-CA)			AE (|DA-CA|)			SE	*R*^2^
					*μ*	*σ*	med.	*μ*	*σ*	med.		
Sezer and Çarıkçıoğlu [84]	Turkish	980	6–15	London Atlas [10]	+ 0.09	0.57	–	0.43	0.36	–	–	–
Baylis and Bassed [63]	New Zealander	875	5–18	London Atlas [10]	+ 0.40/ + 0.74	0.77/0.79	–	0.76/0.93	0.50/0.61	–	–	–
				Schour and Massler [8]	− 0.39/− 0.03	0.77/0.83	–	0.86/0.84	0.48/0.52	–	–	–
				Blenkin and Taylor [11]	+ 0.07/ + 0.34	0.77/0.94	–	0.77/0.89	0.47/0.55	–	–	–
Pavlović et al. [85]	Portuguese	736	3–24	London Atlas [10]	+ 0.09	1.45	–	–	–	–	–	–
Ghafari et al. [86]	Iranian	339	6–16	London Atlas [10]	+ 0.09	–	–	0.60	0.57	–	–	0.92
Alsudairi and AlQahatni [87]	Saudi	400	6–16	London Atlas [10]	− 0.59	1.46	–	1.16	1.05	–	–	–
Sharma and Wadhwan [88]	Indian	335	5–16	London Atlas [10]	+ 0.03	0.70	–	0.54	0.44	–	–	–
Gelbrich et al. [89]	German	500	6–16	London Atlas [10]	+ 0.30	–	–	0.90	–	–	–	–
da Silveira et al. [90]	Brazilian	288	5–23	London Atlas [10]	+ 0.55	–	–	1.43	–	–	–	–
Alkandiri et al. [91]	Kuwaiti	375	5–15	London Atlas [10]	+ 0.19/+ 0.60	–	–	–	–	–	–	–

Regarding the staging methods, the one proposed by Nolla [12] was used in eight different studies, as seen in Table 4, showing mean residual errors between − 1.12 and + 0.54 years, and standard deviation values between 0.23 and 3.30. The mean absolute errors ranged from 0.66 to 1.10 years. Most of the studies were focused on subjects between five and 15 years of age, although Berkvens et al. [64] conducted a study on subjects aged up to 30.

**Table 4:** Evaluation of the Nolla method [12]. When two values are given, they correspond to the metrics obtained on males/females

Evaluation work	Population	*n*	Age	E (DA-CA)			AE (|DA-CA|)			SE	*R*^2^
				*μ*	*σ*	med.	*μ*	*σ*	med.		
Kumaresan et al. [82]	Malaysian	426	5–15	+ 0.54	1.31	–	1.10	–	–	–	–
Berkvens et al. [64]	Canadian	361	8–30	–	–	–	–	–	–	–	0.88
Hedge et al. [92]	Indian	1200	5–15	− 0.13/− 0.30	0.81/0.82	–	–	–	–	–	–
Melo and Ata-Ali [93]	Spanish	2541	7–21	− 0.27/− 0.16	0.50/0.23	–	–	–	–	–	–
da Luz et al. [94]	Brazilian	930	8–15	+ 0.09/ + 0.03	0.97/1.27	–	0.80/0.99	0.56/0.79	0.74/0.75	–	–
	Croatian	924	8–15	− 0.13/− 0.44	1.43/1.44	–	1.08/1.07	0.94/1.05	0.85/0.75	–	–
AlQadi et al. [95]	Yemeni	358	8–16	− 0.59/− 0.78	1.28/1.21	–	–	–	–	–	0.77/0.79
Yassin et al. [96]	Saudi	458	5–11	− 1.05/− 1.12	3.07/3.30	–	–	–	–	–	–
Han et al. [97]	Chinese	2000	5–15	+ 0.18/− 0.02	1.22/1.27	–	0.66/0.77	–	–	–	–
Koç et al. [98]	Turkish	916	6–14	− 0.49	1.02	–	0.89	–	–	–	–

The method developed by Demirjian et al. [13] is perhaps one of the most studied approaches for the estimation of dental age. In the analysed period of time, a set of 40 studies using Demirjian et al.’s method [13] reported any of the required metrics in different populations, as shown in Table 5. The range of ages was also wider than in the case of Nolla’s method [12], with subjects ranging from two to 30, although most of them focused on the interval between five and 23. Regarding the obtained results, a clear overestimation can be seen, being the mean errors between − 0.58 and + 2.13 years. Absolute errors indicate that the error magnitude lies between 0.13 and 1.48 years, while the reported *R*^2^ values were over 0.60 in any case.

**Table 5:** Evaluation of the Demirjian et al.’s method [13] and revisited Demirjian’s method [99]. When two values are given, they correspond to the metrics obtained on males/females

Evaluation work	Population	*n*	Age	E (DA-CA)			AE (|DA-CA|)			SE	*R*^2^
				*μ*	*σ*	med.	*μ*	*σ*	med.		
Gelbrich et al. [89]	German	500	6–16	+ 0.11	–	–	0.97	–	–	–	–
Berkvens et al. [64]	Canadian	361	8–30	–	–	–	–	–	–	–	0.87
Hedge et al. [92]	Indian	1200	5–15	+ 0.19/+ 0.11	0.80/0.81	–	–	–	–	–	–
Melo and Ata-Ali [93]	Spanish	2541	7–21	+ 0.99/ + 0.72	0.39/0.56	–	–	–	–	–	–
Yassin et al. [96]	Saudi	627	5–12	+ 0.37/ + 0.69	1.49/1.17	–	–	–	–	–	–
Amanullah et al. [100]	Pakistani	300	9–26	–	–	–	–	–	–	–	0.64/0.60
Duangto et al. [101]	Thai	1134	5–15	+ 0.11/ + 0.10	–	–	–	–	–	0.87/0.73	–
Cavrić et al. [102]	Motswana	1760	6–23	+ 1.25/ + 0.72	1.11/1.02	–	1.36/0.96	0.96/0.80	–	–	–
Chiam et al. [103]	Australian	230	2–15	− 0.24/ + 0.04	0.84/0.82	–	–	–	–	–	–
Bunyarit et al. [104]	Malaysian	1236	5–16	+ 0.05/+ 0.04	0.83/0.89	–	1.24	–	–	–	–
Alsaffar et al. [105]	Maltese	200	4–26	+ 0.02/− 0.02	–	–	–	–	–	–	–
Saade et al. [106]	Lebanese	260	8–17	+ 0.74/ + 0.86	1.38/1.04	–	–	–	–	–	–
Kelmendi et al. [107]	Kosovar	1022	5–14	+ 0.20/+ 0.43	0.80/0.76	–	0.65/0.67	0.51/0.56	–	–	–
Almotairy et al. [108]	Sweedish	107	9–11	+ 1.08	0.84		–	–	–	–	–
Agrawal et al. [109]	British	150	8–19	+ 1.51/ + 2.13	0.96/1.35	–	–	–	–	–	–
Esan et al. [110]	South African	540	5–16	+ 0.85/ + 1.00	–	–	1.10/1.10	–	–	–	–
Sobieska et al. [111]	Polish	1002	4–17	− 0.32	–	–	–	–	–	–	–
Nemsi et al. [112]	Tunisian	500	5–15	− 0.38	0.93	–	0.77	0.64	–	–	–
Al-Balushi et al. [113]	Omani	485	4–17	+ 0.10/ + 0.05	–	–	–	–	–	–	–
Kumagai et al. [114]	Japanese	256	4–20	+ 0.09/+ 0.07	–	–	1.07/1.00	–	–	–	–
Liu et al. [115]	Chinese	2519	8–23	− 0.02	–	–	0.13	–	–	2.03	0.74
Khdairi et al. [116]	German	1260	5–17	+ 0.27/ + 0.41	0.87/0.84	–	–	–	–	–	–
Metsäniitty et al. [117]	Somali	803	3–23	+ 0.26/+ 0.29	–	–	1.03/1.11	–	–	–	–
Ozveren et al.[118]	Turkish	766	6–15	+ 1.04/ + 0.87	0.95/0.92	–	–	–	–	–	–
Lopes et al. [119]	Brazilian	403	7–13	+ 1.49/ + 1.47	–	–	–	–	–	–	
Birchler et al. [120]	Finnish	100	6–15	+ 0.34	0.87	+ 0.32	–	–	–	–	–
Ranasinghe et al. [121]	Sri Lankan	688	8–17	+ 0.19	0.87	–	0.70	0.55	–	–	–
Kermani et al. [122]	Iranian	158	5–13	+ 0.67/ + 1.44	0.92/0.88	-	0.86/1.48	0.75/0.81	–	–	–
Ginzelova et al. [123]	Czech	579	3–16	+ 0.06	1.60	–	–	–	–	–	–
Alqadi et al. [124]	Yemeni	357	8–16	− 0.58	1.25	-	-	-	-	-	0.71/0.78
Moness et al. [125]	Egyptian	160	3–10	+ 0.47/ + 0.33	–	–	–	–	–	–	–
Shen et al. [126]	Taiwanese	799	8–16	+ 0.12/ + 0.21	0.80/0.95	–	–	–	–	–	–
Memorando [127]	Filipino	384	9–23	–	–	–	1.05/1.06	–	–	–	–
Pinchi et al. [128]	Italian	752	3–16	+ 0.41	0.88	–	–	–	0.67	–	–
Subedi et al. [129]	Nepalese	352	5–23	+ 0.01/ + 0.00	–	–	1.02/1.23	0.86/1.42	–	–	0.94/0.89
Galibourg et al. [130]	French	3570	2–24	+ 0.71	0.07	–	1.11	0.05	–	–	0.82
Karimi et al. [131]	Kuwaiti	1393	3–26	− 0.14/ + 0.33	1.23/0.84	–	–	–	–	–	–
Rodríguez et al. [132]	Mexican	182	6–15	+ 0.70/ + 0.51	–	–	1.00/1.06	–	–	–	–
Jayaraman et al. [133]	American	600	6–17	− 0.04/− 0.07	0.51/0.41	–	–	–	–	–	–
	(Hispanic)										
Shi et al. [134]	Tibetan	1951	4-15	− 0.46/− 0.48	1.09/1.04	–	0.96/0.96	–	–	–	–

The modified Demirjian’s method developed by Willems et al. [14] led to numerous studies focused on testing its applicability in different populations. In this review, a set of 28 studies was analysed (Table 6). On average, the method was applied to a narrower age range, working most of the authors in the range between five and 16 years of age. Although more investigations that show overestimation than underestimation—13 vs. 12, respectively—, this trend is much less noticeable than in the case of the method by Demirjian et al. [13]. The absolute errors also tended to decrease with this method, as the values lied between 0.61 and 1.16 years. Bedek et al. [15] proposed a modification of Willems et al.’s method [14], which was evaluated in an Indian population by Sheriff et al. [65], as it can be seen in Table 7. The results showed a notable underestimation (up to − 0.55 years of mean error), but the low standard deviation values (0.05 to 0.06 years) indicated that the error was consistent between all subjects.

**Table 6:** Evaluation of the Willems et al,’s method [14] to estimate age in subadults. When two values are given, they correspond to the metrics obtained in males/females

Evaluation work	Population	*n*	Age	E (DA-CA)			AE (|DA-CA|)			SE	*R*^2^
				*μ*	*σ*	med.	*μ*	*σ*	med.		
Kumaresan et al. [82]	Malaysian	426	5–15	+ 0.54	1.28	–	0.99	–	–	–	–
Gelbrich et al. [89]	German	500	6–16	+ 0.38	–	–	0.88	–	–	–	–
Hegde et al. [92]	Indian	1200	5–15	+ 0.09/ + 0.08	0.80/0.80	–	–	–	–	–	–
Duangto et al. [101]	Thai	1134	5–15	− 0.37/− 0.39	–	–	–	–	–	0.91/0.93	–
Saade et al. [106]	Lebanese	260	8–17	+ 0.18/ + 0.08	1.24/1.12	–	–	–	–	–	–
Sobieska et al. [111]	Polish	1002	4–17	− 0.38	–	–	–	–	–	–	–
Agrawal et al. [109]	Nepali	150	8–19	− 0.80/− 1.23	1.13/1.45	–	–	–	–	–	–
Kelmendi et al. [107]	Kosovar	1022	5–14	− 0.14/− 0.24	0.77/0.75	–	0.61/0.64	0.49/0.46	–	–	–
Almotairy et al. [108]	Sweedish	107	9–11	+ 0.46	0.83	–	–	–	–	–	–
Nemsi et al. [112]	Tunisian	500	5–15	− 0.54	3.14	–	1.05	3.01	–	–	–
Ranasinghe et al. [121]	Sri Lankan	688	8–17	− 0.38	0.84	–	0.69	0.61	–	–	–
Shen et al. [126]	Taiwanese	799	8–16	− 0.22/ + 0.12	0.80/0.92	–	–	–	–	–	–
Pinchi et al. [128]	Italian	752	3–16	− 0.25	1.02	–	–	–	0.76	–	–
Galibourg et al. [130]	French	3605	2–24	+ 0.22	0.08	–	0.93	0.04	–	–	0.87
Rodríguez et al. [132]	Mexican	182	6–15	+ 0.05/ + 0.00	–	–	0.66/0.99	–	–	–	–
Shi et al. [134]	Tibetan	1951	4–15	− 0.84/− 1.00	1.03/1.04	–	1.06/1.16	–	–	–	–
Lauc et al. [135]	Bosnian	776	7–15	+ 0.57/ + 0.48	1.06/1.12	–	–	–	–	–	–
Marinkovic et al. [136]	Serbian	423	5–15	+ 0.63/ + 0.58	0.95/0.94	–	–	–	–	–	–
Willems et al. [137]	South African	986	4–15	− 0.06	0.86	+ 0.00	0.69	0.52	0.57	–	–
Metsäniitty et al. [138]	Somali	808	4–18	− 0.09	1.01	–	0.78	0.65	–	–	–
Kurniawan et al. [139]	Indonesian	110	6–14	+ 0.15	0.92	–	–	–	–	–	–
Ortega–Pertuz and Piña D’Abreu [140]	Venezuelan	458	6–18	+ 0.71	1.23	–	–	–	–	–	–
Paz–Cortés et al. [77]	Spanish	604	4–13	+ 0.26	0.91	–	–	–	–	–	–
Pan et al. [141]	Chinese	2367	5–16	− 0.07/− 0.24	0.92/1.03	–	0.70/0.79	–	–	–	–
Çarıkçıoğlu and Değirmenci [142]	Turkish	1024	6–16	+ 0.23	0.80	–	0.66	0.51	–	–	–
Rocha et al. [143]	Brazilian	1000	6–16	+ 0.18/− 0.01	–	–	0.78/0.79	–	–	–	–
Yassin et al. [144]	Saudi	1206	4–14	− 0.39	1.48	–	–	–	–	–	–
Cadenas et al. [145]	Kenyan	1083	3–24	+ 0.00	1.30	+ 0.00	0.97	0.86	0.76	–	–

**Table 7:** Evaluation of Bedek et al.’s method [15] to estimate age in subadults. When two values are given, they correspond to the metrics obtained in males/females

Evaluation work	Population	*n*	Age	E (DA-CA)			AE (|DA-CA|)			SE	*R*^2^
				*μ*	*σ*	med.	*μ*	*σ*	med.		
Sheriff et al. [65]	Indian	650	7–15	− 0.55/− 0.36	0.06/0.05	–	–	–	–	–	–

The modification of the Demirjian et al.’s method [13] proposed by Chaillet and Willems [16] was applied to four different samples in the collected studies, as presented in Table 8. The range of application, however, is narrower than in the Demirjian’s applications, as the subjects were in every case younger than 18. Unlike the systematic overestimation of the Demirjian et al.’s method [13], Chaillet and Willems’ [16] tended to underestimate age, with mean errors between − 2.79 and − 0.07 years. Absolute errors ranged from 0.66 to 1.14 years on average, with standard deviations between 0.49 and 0.52 years.

**Table 8:** Evaluation of Chaillet and Willems’ method [16] to estimate age in subadults. When two values are given, they correspond to the metrics obtained in males/females

Evaluation work	Population	*n*	Age	E (DA-CA)			AE (|DA-CA|)			SE	*R*^2^
				*μ*	*σ*	med.	*μ*	*σ*	med.		
Kelmendi et al. [107]	Kosovar	1022	5–14	− 0.24/− 0.35	0.85/0.74	–	0.72/0.66	0.52/0.49	–	–	–
Rodríguez et al. [132]	Mexican	182	6–15	+ 0.07/− 0.39	–	–	0.78/1.14	–	–	–	–
Hegde et al. [146]	Indian	1200	5–15	− 0.12	0.69	–	–	–	–	–	0.55
Bunyarit et al. [147]	Malaysian	1569	5–18	− 2.09/− 2.79	0.90/0.99	–	–	–	–	–	–

Finally, the modified Demirjian’s method developed by Blenkin and Evans [17] was applied to two different populations of subjects aged six to 17 (Table 9), yielding errors with mean values ranging from − 0.05 to − 0.55 years and standard deviations up to 1.04 years. The absolute errors ranged from 0.61 to 0.91 years on average.

**Table 9:** Evaluation of Blenkin and Evans’ method [17] to estimate age in subadults

Evaluation work	Population	*n*	Age	E (DA-CA)			AE (|DA-CA|)			SE	*R*^2^
				*μ*	*σ*	med.	*μ*	*σ*	med.		
Ranasinghe et al. [121]	Sri Lankan	688	8–17	− 0.55	1.04	–	0.91	0.75	–	–	–
Çarıkçıoğlu and Değirmenci [142]	Turkish	1024	6–16	− 0.05	0.77	–	0.61	0.47	–	–	–

The tooth staging criteria proposed by Gleiser and Hunt [18] led to the development of several methods, such as those proposed by Moorrees et al. [19] and Kohler et al. [20]. Six studies applied the former in different samples of subjects aged from three to 30, as seen in Table 10, with mean errors between − 1.01 and + 0.34 and so a tendency to underestimating the age. In absolute terms, the error ranged between 0.63 and 1.42 years. On the other hand, Kohler et al.’s method [20] was applied to two different samples of subjects up to 24 years of age, reaching systematic underestimations in both cases and mean absolute errors up to 2 years.

**Table 10:** Evaluation of the methods based on Gleiser and Gunt criteria [18]. When two values are given, they correspond to the metrics obtained on males/females

Evaluation work	Population	*n*	Age	Estimation method	E (DA-CA)			AE (|DA-CA|)			SE	*R*^2^
					*μ*	*σ*	med.	*μ*	*σ*	med.		
Alkandiri et al. [91]	Kuwaiti	375	5–15	Moorrees et al. [19]	− 0.89/− 1.01	–	–	–	–	–	–	–
Berkvens et al. [64]	Canadian	361	8–30	Moorrees et al. [19]	–	–	–	–	–	–	–	0.09
Metsäniitty et al. [117]	Somali	803	3–23	Kohler et al. [20]	− 0.29/− 0.30	–	–	1.39/1.30	–	–	–	–
Rodríguez et al. [132]	Mexican	182	6–15	Moorrees et al. [19]	− 0.03/ + 0.34	–	–	0.63/0.98	–	–	–	–
Jayaraman et al. [133]	Hispanic American	600	6–17	Moorrees et al. [19]	− 0.07	0.45	–	–	–	–	–	–
Fulton and Liversidge [148]	British	940	3–16	Moorrees et al. [19]	− 0.22	2.08	–	1.42	–	–	–	–
Štepanovskỳ et al. [149]	Czech	976	3–20	Moorrees et al. [19]	–	–	–	0.64	–	–	–	–
Sartori et al. [150]	Brazilian	1062	15—24	Kohler et al. [20]	− 1.30	2.10	–	2.00	1.50	–	–	–

The widely used tooth staging method proposed by Liliequist and Lundberg [21] was used in two of the retrieved studies, in Brazilian and Croatian populations, respectively. As it can be seen in Table 11, the method led to an age underestimation in both cases, though it was more noticeable in the former, with a mean error of − 0.58 years. Absolute errors were very similar in both studies, with mean values of 0.97 and 0.99 years and median values of 0.83 and 0.81, respectively.

**Table 11:** Evaluation of Liliequist and Lundberg’s method [21]

Evaluation work	Population	*n*	Age	E (DA-CA)			AE (|DA-CA|)			SE	*R*^2^
				*μ*	*σ*	med.	*μ*	*σ*	med.		
Benedicto et al. [83]	Brazilian	1059	8–16	− 0.58	–	–	0.97	–	0.83	–	–
Da Luz et al. [94]	Croatian	924	8–15	− 0.05	1.27	–	0.99	0.81	0.81	–	–

The method proposed by De Tobel et al. [22], which mixed both Demirjian et al. [13] and Kohler et al.’s [20] staging systems, was applied to a Belgian and Dutch sample of subjects aged 14 to 26 as presented in Table 12. The authors reported mean errors closer to zero and thus an equal tendency to overestimating or underestimating age, while the mean absolute error was between 1.70 and 2.00 years.

**Table 12:** Evaluation of De Tobel et al.’s method [22] to estimate age in subadults and young adults

Evaluation work	Population	*n*	Age	E (DA-CA)			AE (|DA-CA|)			SE	*R*^2^
				*μ*	*σ*	med.	*μ*	*σ*	med.		
De Tobel et al. [151]	Belgian and Dutch	309	14–26	+ 0.00/ + 0.10	–	–	1.70/2.00	–	–	–	–

As seen in Table 13, Cameriere et al.’s method [23] based on the evaluation of open apices led to a systematic underestimation in most evaluated populations—13 out of 14 reporting the residuals—, with a maximum mean error of − 1.36 years, although it is noticeable that in the Rivera et al. study [66], the mean error is negative while the median error of male subjects is positive. The absolute errors ranged between 0.57 and 1.60 years on average.

**Table 13:** Evaluation of Cameriere’s method [23] for age estimation in subadults. When two values are given, they correspond to the metrics obtained on males/females

Evaluation work	Population	*n*	Age	E (DA-CA)			AE (|DA-CA|)			SE	*R*^2^
				*μ*	*σ*	med.	*μ*	*σ*	med.		
Kumaresan et al. [82]	Malaysian	426	5–15	− 0.41	1.08	–	0.89	–	–	–	–
Sharma and Wadhwan [88]	Indian	335	5–16	− 0.60	1.32	–	1.11	0.93	–	–	–
da Luz et al. [94]	Brazilian	930	8–15	− 1.08/− 1.03	1.13/1.14	–	1.27/1.26	0.91/0.88	1.06/1.13	–	–
	Croatian	924	8–15	− 1.20/− 1.19	1.25/1.36	–	1.38/1.39	1.05/1.16	1.16/1.11	–	–
Marinkovic et al. [136]	Serbian	423	5–15	− 0.38/− 0.38	0.93/0.92	–	–	–	–	–	–
Çarıkçıoğlu and Değirmenci [142]	Turkish	1024	6–16	− 0.51	0.90	–	0.77	0.68	–	–	–
Rivera et al. [66]	Colombian	526	6–14	− 0.08	0.69	+ 0.15/− 0.28	0.57/0.57	0.38/0.41	–	–	–
Cameriere et al. [152]	Italian	2630	4–17	–	–	–	0.72/0.73	0.60/0.61	–	–	–
Santana et al. [153]	American - Indian	57	6–17	− 1.36	1.47	− 1.02	1.55	–	1.05	–	–
	American - European	173	6–17	− 1.24	1.72	− 1.02	1.60	–	1.21	–	–
	American - Hispanic	130	6–17	− 1.24	1.52	− 0.96	1.48	–	1.15	–	–
Halilah et al. [154]	German	1000	5–16	− 0.64/− 0.38	0.91/0.88	− 0.36/− 0.34	0.92/0.76	–	0.79/0.64	–	0.85/0.83
Angelakopoulos et al. [155]	South African - Black	970	6–14	–	–	–	0.70	–	–	–	0.82
	South African - White	974	6–14	–	–	–	0.58	–	–	–	0.874
AlShahrani et al. [156]	Saudi	788	6–16	− 0.26	1.47	–	–	–	–	–	0.49
Różyło–Kalinowska et al. [157]	Polish	121	5–13	+ 0.17/+ 0.18	0.86/0.96	–	0.73/0.77	0.48/0.60	–	–	–
Shen et al. [158]	Chinese	748	5–13	− 0.39	0.04	–	0.81	0.02	–	–	–

The proposed approaches for estimating chronological age in adults produced systematically worse results than their children-orientated counterparts, and the available studies are much scarcer. Moreover, the studies that apply adult-based methods tended to report mostly the standard error and *R*^2^ values instead of the residual and absolute error measurements, in opposition to the previously presented methods. In this regard, 35 studies were collected related to the evaluation of metric methods based on a set of linear and volumetric tooth analysis, as seen in Table 14. The most common approach is the pulp-to-tooth linear, area, and volumetric ratio (PTLR, PTAR, PRVR), used in eight works each, and the tooth-to-crown index (TCI) and the pulp-to-crown volume ratio (PCVR), each one applied in three studies. It is also worth noting that most of these works relied on 3D images (such as CT-based records) instead of flat X-rays, as they allow the volume of the different tooth structures to be analysed accurately.

**Table 14:** Evaluation of methods based on evaluation of linear and/or volumetric dental measurements. When two values are given, they correspond to the metrics obtained in males/females. *TCI*, tooth coronal indexes; *PLR*, pulp linear ratio; *PTLR*, pulp-to-tooth linear ratio; *PTAR*, pulp-to-tooth area ratio; *PTVR*, pulp-to-tooth volume ratio; *PV*, pulp volume; *PCLR*, pulp-to-crown linear ratio; *PCVR*, pulp-to-crown volume ratio; *PEVR*, pulp-to-enamel volume ratio; *PDVR*, pulp-to-dentin volume ratio

Evaluation work	Measurement	Population	*n*	Age	E (DA-CA)			AE(|DA-CA|)			SE	*R*^2^
					*μ*	*σ*	med.	*μ*	*σ*	med.		
Li et al. [67]	PTLR (Kvaal et al.)	Chinese	360	20–65	+ 8.6	11.80	–	–	–	–	11.40	0.23
Roh et al. [68]	PTLR (Kvaal et al.)	South Korean	266	21–69	− 9.01	11.58	–	12.58	7.54	–	10.70	0.47
Dabbaghi and Kazemi [69]	PTAR (Cameriere et al.)	Iranian	153	13-70	+ 8.83	0.52	–	–	–	–	11.06	0.54
Cameriere et al. [70]	PTAR (Cameriere et al.)	Turkish, Italian, Portuguese, Japanese and Mexican	891	20–86	–	–	–	2.43/2.49	–	–	–	–
Jain et al. [159]	TCI, PCLR	Indian	180	15–70	+ 1.34	–	–	–	–	–	–	0.83
Akay et al. [160]	TCI	British	250	18–60	–	–	–	–	–	–	10.37	0.28
Gok et al. [161]	TCI	Turkish	9059	15–40	–	–	–	–	–	–	7.25/7.02	0.08/0.06
Herianti et al. [162]	PLR	Indonesian	113	11–60	–	–	–	–	–	–	4.66	0.88
Hisham et al. [163]	PTLR (Kvaal et al.)	Malaysian	718	16–80	–	–	–	–	–	–	15.29	0.06
Akay et al. [164]	PTLR (Kvaal et al.)	Turkish	134	16-71	–	–	–	–	–	–	–	0.39
	PTVR				–	–	–	–	–	–	–	0.52
de Miranda et al. [165]	PTLR (Kvaal et al.)	Brazilian	320	20-59	–	–	–	6.81	4.06	–	–	–
	PTAR (Cameriere et al.)				–	–	–	7.55	5.35	–	–	–
Limdiwala et al. [166]	PTLR (Kvaal et al.)	Indian	160	18–62	–	–	–	–	–	–	12.47	0.01
Vossoughi et al. [167]	PTLR (Kvaal et al.)	Iranian	240	20–85	–	–	–	7.53	5.34	–	9.22	0.68
Farhadian et al. [168]	PTLR, PTAR	Iranian	300	14–60	–	–	–	4.12	–	–	–	0.86
Jambunath et al. [169]	PTAR (Cameriere et al.)	Indian	250	18–75	–	–	–	7.50	–	–	–	0.48
Lee et al. [170]	PTAR (Cameriere et al.)	Korean	402	20–78	–	–	–	–	–	–	10.40	0.62
Kumar et al. [171]	PTAR	Indian	400	14–60	–	–	–	–	–	–	12.00	0.17
Dehghani et al. [172]	PTAR	Iranian	271	16–64	–	–	–	6.07	1.70	–	–	–
Lee et al. [173]	PTVR	South Korean	205	20–77	–	–	–	–	–	–	–	0.52
Haghanifar et al. [174]	PTVR	Iranian	377	20–69	–	–	–	–	–	–	7.21/7.68	0.55/0.50
Asif et al. [175]	PTVR	Malaysian	300	16–65	–	–	–	5.66	–	–	5.84	–
Zhang et al. [176]	PTVR	Chinese	392	16–76	–	–	–	6.80/7.90	–	–	–	0.67/0.63
Muralidhar et al. [177]	PTVR	Indian	100	19–70	–	–	–	–	–	–	7.21/7.68	0.55/0.50
Pires et al. [78]	PTVR	Portuguese	158	21–80	− 21.36	16.51	–	25.85	–	–	–	–
Kazmi et al. [178]	PTVR	Pakistani	719	15–65	–	–	–	–	–	–	–	0.46
Helmy et al. [179]	PV	Egyptian	187	21–50	–	–	–	4.61	–	–	5.87	0.48
Ge et al. [180]	PV	Chinese	250	16–63	–	–	–	–	–	6.26	–	0.70
Singal et al. [181]	PCLR	Indian	416	15–54	− 0.29/− 0.28	–	–	–	–	–	–	0.97
Elgazzar et al. [182]	PCVR	Egyptian	200	15–60	− 0.11	–	–	–	–	–	–	0.95
Molina et al. [183]	PCVR	Spanish	107	14–70	–	–	–	8.00	5.7	–	–	0.37
Asif et al. [184]	PCVR	Malaysian	110	16–65	–	–	–	–	–	–	6.83	0.78
Zhang et al. [185]	PEVR	Chinese	414	20–64	–	–	–	8.41	–	–	–	0.42
Nemsi et al. [186]	PDVR	Tunisian	120	22–67	–	–	–	–	–	–	7.06	–

Huge variability in the results reported by these studies is observed. For example, the mean absolute error varied not only depending on the measurement but also across the studies using the same measurement (from 5.66 to 25.85 years in the case of PTVR). This can also be seen in the standard error metric, which lied between 4.66 and 15.29 years. In terms of variance explained, the models moved between 1 and 97%. The specific method of Kvaal et al. [26], which is based on pulp-to-tooth linear ratios, yielded very different behaviour in the available studies. For example, while the study by Li et al. [67] reported a noticeable overestimation, the work by Roh et al. [68] pointed out a great underestimation, both of them evaluated in samples of adults aged around 20 to 70. In the same way, studies published on the evaluation of the Cameriere et al.’s method [30] based on the pulp-to-tooth area ratio focused on the statistical analysis of the method and reported limited information regarding the residual error of the model. Only Dabbaghi and Kazemi [69] reported the mean error, pointing out an overestimation of 8.83 years. Furthermore, it is noticeable that the lowest absolute errors were obtained in a multi-ethnic sample [70].

Methods that have associated radiographic visibility of several oral structures with chronological age usually do not aim to estimate a numerical age value, so only two of them reported estimation error metrics. As shown in Table 15, the study of Chaudhary and Liversidge [71] pointed out an overall overestimate of 7.21 years for males and 6.87 years for females, being the mean absolute error of 7.91 and 7.74 years in the same two scenarios. On the other hand, Timme et al. [72] did not report the error metrics, but a standard error of 3.55 years and the percentage of explained variance (69%).

**Table 15:** Evaluation of methods to estimate the age of adults based on the radiographic visibility of several structures. When two values are given, they correspond to the metrics obtained in males/females. *PL*, periodontal ligament; *RP*, root pulp

Evaluation work	Observed structure	Population	*n*	Age	E (DA-CA)			AE (|DA-CA|)			SE	*R*^2^
					*μ*	*σ*	med.	*μ*	*σ*	med.		
Chaudhary and Liversidge [71]	PL	British	163	16–45	+ 7.21/ + 6.87	5.16/5.83	–	7.91/7.74	–	–	–	–
Timme et al. [72]	PL and RP	German	1245	15–40	–	–	–	–	–	–	3.55	0.69

Methods based on the evaluation of degenerative tooth changes, based on Gustafson’s criteria [35], are summarised in Table 16. The results show again a high degree of variability, with standard errors reaching a minimum of 0.69 and a maximum of 10.92 years, even if in both cases the Gustafson criteria are assessed in a population of the same ethnic origin. Only two studies reported the mean absolute error, with very different values in each one (3.34 and 3.68 years in the first method and 11.08 years in the second). Finally, *R*^2^ values were in the range 0.23–0.80.

**Table 16:** Evaluation of methods for estimating age in adults based on Gustafson’s criteria [35]. When two values are given, they correspond to the metrics obtained on males/females

Evaluation work	Population	*n*	Age	Estimation method	E (DA-CA)			AE (|DA-CA|)			SE	*R*^2^
					*μ*	*σ*	med.	*μ*	*σ*	med.		
Timme [37]	German	2346	15–70	Olze [36]	–	–	–	–	–	–	6.75	0.80
Akay [160]	British	250	18–60	Olze [36]	–	–	–	–	–	–	5.72	0.79
Sonjaya [187]	Malaysian	400	18–74	Gustafson [35]	− 0.17	0.98	–	–	–	–	0.69	0.60
Si [188]	Chinese	1300	15–40	Olze [36]	− 0.47/− 0.76	–	–	3.43/3.68	–	–	4.75	0.68
Dezem [189]	Brazilian	503	15–70	Olze [36]	–	–	–	11.08	–	–	–	0.23
				Timme [37]	–	–	–	11.08	–	–	–	0.23
Koh [190]	Malaysian	284	20–70	Olze [36]	–	–	–	–	–	–	10.92	0.39

As mentioned in “Age estimation on other radiologically observable structures” section, the estimation of chronological age was also approached by mandibular bone analysis, specifically by measuring ramus length [38] and gonial angle [39]. The former produced a model that represented 62% of the data variance, while the latter led to an absolute error of 13.98 years. As it can be seen in Table 17, both studies reported different metrics, so they are not directly comparable.

**Table 17:** Evaluation of methods to estimate the age based on the measurement of the mandible

Evaluation work	Measurement	Population	*n*	Age	E (DA-CA)			AE (|DA-CA|)			SE	*R*^2^
					*μ*	*σ*	med.	*μ*	*σ*	med.		
Motawei et al. [38]	Ramus length	Egyptian	213	7–58	–	–	–	–	–	–	–	0.62
Acharya [39]	Gonial angle	Indian	100	18–89	–	–	–	13.98	–	–	15.84	–

**Fig. 4:**
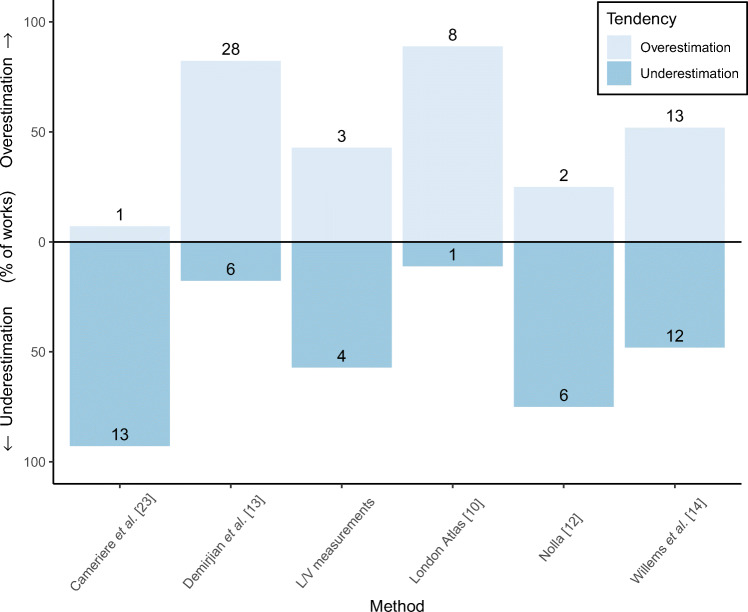
Underestimation or overestimation produced by the methods for numeric age estimation. Only those methods reporting the mean error in at least five studies were included. The methods based on linear and volumetric measurements of the teeth (L/V measurements in the figure) were grouped for a better representativity

The most widely used methods for numeric age estimation were jointly analysed regarding the obtained underestimation or overestimation. As it can be seen in Fig. 4, two of the six methods showed a clear pattern of overestimation, namely those proposed by Demirjian et al. [13] and the London Atlas [10]. On the other hand, the methods developed by Cameriere et al. [23] and Nolla [12] led to a systematic underestimation of age. Finally, the methods based on linear and volumetric measurements of the teeth, as well as that proposed by Willems and Chaillet [14] yielded a more balanced performance, with almost the same number of studies underestimating and overestimating age.

As mentioned in the previous section, some age estimation methods were adapted to work as a binary classifier for detecting people younger or older than the legal age. The results obtained in this regard are presented in Table 18. First, the methods based on tooth eruption presented by Haavikko [7] and Olze et al. [40] were assessed in the problem of 14-year-old detection. The former led to accuracy between 78 and 81%, while the latter yielded better performance, with 83 to 86%. Also, the method proposed by Olze et al. showed a more balanced behaviour, with similar sensitivity and specificity values.

**Table 18:** Evaluation of age thresholding methods. When two values are given, they correspond to males/females

Method	Method work	Evaluation work	Population	*n*	Age	Target	Accuracy	Sensitivity	Specificity
Tooth eruption	Haavikko [7]	Pinchi et al. [75]	Italian	501	11–15	14	78/81	88/72	70/86
	Olze et al. [40]	Thomas et al. [191]	Indian	640	10–18	14	86/83	89/89	84/78
Atlas-based	London Atlas [10]	De Moraes et al. [73]	Brazilian	1200	16–21	18	80	92	56
Nolla development stages	Nolla [12]	Pereira et al. [45]	Portuguese	348	12–23	14	82	91	60
						16	87	80	92
						18	90	81	95
						21	84	94	82
		Antunovic et al. [192]	Montenegrin	683	13–24	18	90/87	95/85	84/90
Demirjian development stages	Willems et al. [14]	Pinchi et al. [75]	Italian	501	11–15	14	77/83	74/78	79/86
	Mincer et al. [42]	Mwesigwa et al. [74]	Ugandan	1021	10–22	12	–	93	80
						14	–	97	83
						16	–	69	97
						18	–	88	88
		Pinchi et al. [75]	Italian	501	11–15	14	78/83	80/82	76/84
		Lucas et al. [193]	British	2000	16–26	18	93/91	92/91	98/92
		Friedrich et al. [194]	German	1804	15–24	18	–	74	73
		Márquez–Ruiz [195]	Mexican	135	14–23	18	–	85	71
		Lizarbe et al. [196]	Peruvian	208	14–22	18	86	88	85
		Hassan et al. [197]	Egyptian	350	14–24	18	–	–	92/91
		Sharma et al. [198]	Indian	1062	14–23	18	93	93	92
		Augusto et al. [79]	Portuguese	1104	7–23	12	91	95	81
						14	90	77	93
		Melo et al. [199]	Spanish	1386	10–26	18	94	94	95
Gleiser and Hunt staging system	Moorrees et al. [19]	Pereira et al. [45]	Portuguese	348	12–23	14	83	92	59
						16	87	82	91
						18	90	80	95
						21	84	92	82
	Kohler et al. [20]	Yellapurkar et al. [44]	Indian	404	7–20	14	91	–	–
		Franco et al. [200]	Russian	918	8–23	14	–	87/80	87/90
Root pulp visibility	Olze et al. [31]	Akkaya et al. [201]	Turkish	463	14–34	18	–	89/83	91/67
						21	–	86/73	88/93
		Manthapuri et al. [202]	Indian	760	12–20	16	77/80	61/65	96/97
Cameriere’s method	Cameriere et al. [23]	Pinchi et al. [75]	Italian	501	11–15	14	85/87	77/85	92/88
		Antunovic et al. [192]	Montenegrin	683	13–24	18	93/89	92/82	94/96
		Lizarbe et al. [196]	Peruvian	208	14–22	18	96	96	96
		Hassan et al. [197]	Egyptian	350	14–24 0	18	89	92	84
		Augusto et al. [79]	Portuguese	1104	7–23	12	89	94	74
						14	87	87	88
		Melo et al. [199]	Spanish	1386	10–26	18	96	90	99
		De Luca et al. [203]	Colombian	288	13–22	18	91/94	92/95	91/94
		Cavrić et al. [204]	Motswana	1294	13–23	18	91/92	88/88	95/96
		Zelic et al. [205]	Serbian	598	13–24	18	95/91	96/86	94/98
		Gulsahi et al. [206]	Turkish	293	14–22	18	98/93	95/86	100/100
		Franklin et al. [207]	Australian	143	14–22	18	87/88	90/90	85/88
		Dardouri et al. [208]	Lybian	307	14–22	18	95/94	91/91	100/100
		AlQahtani et al. [76]	Saudi	300	14–22	18	76/72	51/52	100/97
		Balla et al. [209]	Indian	1330	15–22	18	90/88	91/86	90/90
		Cameriere et al. [210]	Chilean	822	11–22	14	86/80	84/81	90/80
						16	80	79	81
						18	83	71	88
		Angelakopoulos et al. [211]	South African	833	14–24	18	90	80	95
		Różyło–Kalinowska et al. [212]	Polish	982	15–24	18	87	85	92
		Kelmendi et al. [213]	Kosovar	1221	12–23	18	97/91	96/83	98/99
		Spinas et al. [214]	Italian	336	15–23	18	86	82	95
		Doğru et al. [215]	Dutch	360	14–22	18	89/83	84/73	95/96
		Balla et al. [216]	Indian	819	11–20	16	88/89	91/90	86/87
		Kumagai et al. [217]	Japanese	276	14–24	18	91/87	89/84	96/93
		Chu et al. [218]	Chinese	840	13–24	18	92	93/94	81/97
		Moukarzel et al. [219]	Lebanese	620	14–23	18	74/79	63/61	89/97
		Ribier et al. [220]	French	431	14–22	18	91/81	95/83	85/79
		Scendoni et al. [221]	Russian	571	14–24	18	97/95	96/93	98/98
		Wang et al. [222]	Chinese	671	10–20	16	89	78	97
		Angelakopoulos et al. [223]	African	1631	13–24	18	83	87	84
			American	861			86	80	91
			Asian	3919			73	61	91
			European	3770			85	80	95
		Yılancı et al. [224]	Turkish	763	10–17	12	–	90/91	78/77
						15	–	93/89	87/78

There is only one study that evaluated an Atlas-based method for binary age classification. Specifically, De Moraes et al. [73] used the London Atlas [10] for classifying dental records according to the 18-year-old threshold. Although the accuracy reached a reasonable value of 80%, the methods were heavily biased, as they produced a very high sensitivity—that is, they correctly detected subjects older than 18— but very low specificity —it only correctly classified half of the subjects younger than 18.

Dental staging methods were used to a greater extent. Regarding the Nolla method [12], it was applied to the Portuguese and Montenegrin populations. In the first, the method obtained accuracies from 82 to 90%, depending on the age threshold, while in the latter the accuracy was 90% for males and 87% for females. It is noticeable that Nolla’s method was highly biased in both directions in the Portuguese population, as it obtained higher sensitivity values with the thresholds of 14 and 21 years, and better specificity values with the age thresholds of 16 and 18.

The Mincer et al.’s method [42] based on the development stages proposed by Demirjian et al. [13] led to maximum accuracies of 91, 90, and 94 % when using the legal age thresholds of 12, 14, and 18 years, respectively. Although the sensitivity and specificity were almost balanced in most studies, there are some cases where a significant bias was observed, such as the study by Mwesigwa et al. [74] with a legal age of 16—sensitivity of 69% vs. specificity of 97%. Pinchi et al. [75] also tested the Willems et al.’s scores [14] in an Italian population, reaching a slightly worse result than in the case of the original scores, especially in the sensitivity values (74–78% vs. 80–82%).

Gleiser and Hunt staging system [18] was also studied on the problem of binary age classification via the derived methods of Moorrees et al. [19] and Kohler et al. [20]. The former was applied in a Portuguese sample with 14, 16, 18, and 21 thresholds, obtaining accuracies from 83 to 90%. Again, the sensitivity and specificity values were highly unbalanced, especially when using the 14-year-old threshold (92% of sensitivity and 59% of specificity). On the other hand, the Kohler et al.’s method reached an accuracy of 91% in an Indian population and sensitivity and specificity values of 87–80% and 87–90%, respectively, in a Russian sample.

The adaptation of Cameriere’s method for legal age classification [62] was by far the most used method for binary age classification. Among all the experiments carried out with this approach, 29 out of the 40 established an age threshold of 18 years. The accuracy values ranged from 72 to 98%, although 35 studies yielded values greater than 80%. As with most methods, there are some cases where a great bias between sensitivity and specificity can be seen, the most significant example being the study by AlQahtani et al. [76], where the sensitivity was 51–52% and the specificity was 100–97%.

Finally, Olze et al.’s [31] method based on the assessment of the root pulp visibility was evaluated in Turkish and Indian samples. Only the latter study reported the accuracy—77% in males and 80% in females—yielding also a notable imbalance between sensitivity and specificity.

**Fig. 5:**
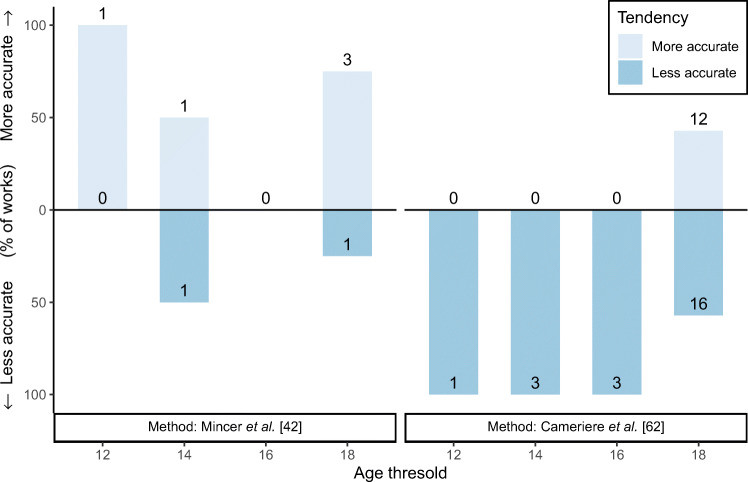
Performance of the methods aimed at age thresholding with respect to an accuracy reference value of 90%. Only those methods used in at least five studies were included

The two most widely applied methods for age thresholding, namely those proposed by Mincer et al. [42] and Cameriere et al. [62], were compared using a reference value of 90% accuracy. As it is shown in Fig. 5, the method of Mincer et al. obtained a performance better than the reference when establishing an age threshold of 12 years and three times out of four with a threshold of 18 years. When the threshold is set to 14 years of age, one study obtained better performance than the reference, and another work reported worse performance. Regarding the Cameriere et al’s method, all studies that set an age threshold of 12, 14, or 16 years reported accuracy values lower than the reference, while studies applying a threshold of 18 years showed a more balanced performance, with 12 studies performing better than the reference and 16 works reporting worse results.

Regarding the automatic approaches proposed for age estimation, each method was tested in a single population. In those aimed at estimating a numerical age value (Table 19), the residual error was systematically closer to zero, being the median between − 0.07 and + 0.12 years. Absolute error varied depending on the age range of the assessed sample. As reported by Vila-Blanco et al. [49], the mean absolute error in a Spanish sample ranged from 0.75 years in subjects younger than 15 years to 2.84 years in subjects younger than 90 years. In this case, the median absolute error was as low as 0.64 years. Comparatively, the work by Čular et al. [46] reported a mean absolute error of 2.28 years in Croatian subjects between 10 and 25 years of age, being 1.75 years on a German sample of subjects aged 5 to 25, as noticed by De Back et al. [48]. Performance was significantly worse in the methods tested in older subjects, as reported by Milošević et al. [55], Zheng et al. [53], and Pham et al. [51], with mean absolute errors of 3.96, 7.17, and 6.97–7.07 years, respectively. On the contrary, the method of Hou et al. [50], which was tested in a large population sample and very wide in terms of subject age (from 0 to 93 years of age), led to a very low mean absolute error, being of 1.64 years. It is also noticeable that the method of Vila-Blanco et al. [54], which relies only on the mandible shape instead of the whole dental image, yielded a mean absolute error of 1.57 years, which is comparable or even better than other methods relying on the whole dental image.

**Table 19:** Results of the main automatic methods for numeric age estimation, evaluated in different populations. When two values are given, they correspond to the metrics obtained in males/females

Evaluation work	Population	*n*	Age	E (DA-CA)			AE (|DA-CA|)			SE	*R*^2^
				*μ*	*σ*	med.	*μ*	*σ*	med.		
Čular et al. [46]	Croatian	203	10–25	–	–	–	2.28	2.17	–	–	–
Štern et al. [47]	Austrian	322	13–25	–	–	–	1.42	1.14	–	–	–
De Back et al. [48]	Germa	> 12,000	5–25	–	–	–	1.75	–	–	–	–
Vila–Blanco et al. [49]	Spanish	2289	4.5–15	–	–	− 0.07	0.75	0.57	0.64	–	0.84
			4.5–20	–	–	+ 0.02	0.89	0.77	0.69	–	0.89
			4.5–25	–	–	+ 0.07	1.17	1.11	0.85	–	0.90
			4.5–30	–	–	+ 0.06	1.43	1.44	0.96	–	0.89
			4.5–40	–	–	+ 0.04	1.80	2.12	1.10	–	0.87
			4.5–89.2	–	–	+ 0.12	2.84	3.75	1.48	–	0.90
Hou et al. [50]	Chinese	27,957	0–93	–	–	–	1.64	–	–	–	–
Pham et al. [51]	South Korean	814	20–70	–	–	–	6.97/7.07	–/0.51	–	–	–
Wallraff et al. [52]	German	14,019	11–20	− 0.30	1.41	–	1.08	–	–	–	–
Zheng et al. [53]	Chinese	180	10–60	–	–	–	7.17	–	–	–	–
Vila–Blanco et al. [54]	Spanish	260	5–17	–	–	–	1.57	1.21	–	2.0	0.80
Milošević et al. [55]	Croatian	4,035	19–90	–	–	–	3.96	–	2.95	–	–

Automatic methods focusing on classifying the subject’s age into a defined set of age ranges led to different results depending on the number of classes, as shown in Table 20. For example, the approaches of De Tobel et al. [56], Merdietio et al. [57], and Banar et al. [58], which proposed to use 10 different development stages, obtained classification accuracies between 51 and 60% and Kappa values between 0.82 and 0.84. It should be noted that these three methods were assessed in the same Belgian sample. On the other hand, Kim et al. [59] and Kahaki et al. [60] reduced the classification problem to five age groups, leading to significantly higher accuracy values of 82 and 90%, respectively.

**Table 20:** Results of the main automatic methods for age group classification, evaluated in different populations

Evaluation work	Population	*n*	Age	#Groups	Accuracy	Others
De Tobel et al. [56]	Belgian	400	–	10	51	Kappa= 82%
Merdietio et al. [57]	Belgian	400	–	10	61	Kappa= 84%
Banar et al. [58]	Belgian	400	7–24	10	60	Kappa= 82%
Kim et al. [59]	South Korean	1586	–	5	90	
Kahaki et al. [60]	Malaysian	456	1–17	5	82	

Finally, Štern et al. [47] and Guo et al. [61] tested their automatic age thresholding approach in Austrian and Chinese populations, respectively, as it can be seen in Table 21. It is worth noting that the latter sample consisted of more than 10,000 OPGs. Guo’s method led to accuracy values between 93 and 96%, depending on the specific age threshold, and was very consistent in terms of sensitivity and specificity. On the other hand, the method by Štern et al. reached a slightly worse accuracy value in the same scenario (85% vs. 93%), and a notable imbalance between sensitivity and specificity is observed.

**Table 21:** Results of the main automatic methods for age thresholding, evaluated in different populations. When two values are given, they correspond to the metrics obtained in males/females

Evaluation work	Population	*n*	Age	Threshold	Accuracy	Sensitivity	Specificity
Štern et al. [47]	Austrian	322	13–25	18	85	75	93
Guo et al. [61]	Chinese	10,257	5–24	14	96	95	96
				16	96	96	96
				18	93	91	94

**Fig. 6:**
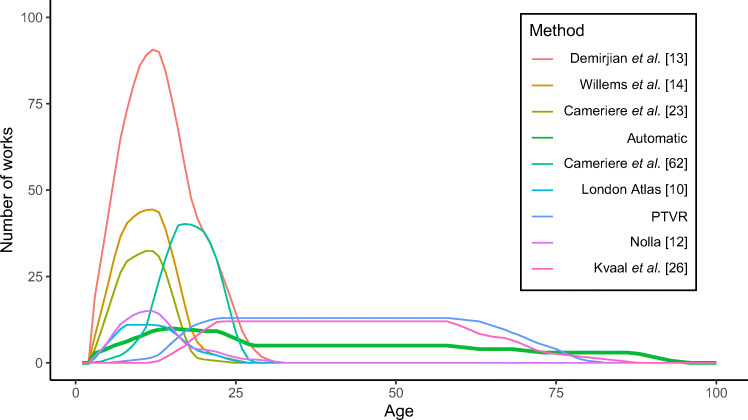
Application of the most widely used methods for age estimation regarding the age of the subjects included in the analysed studies. Only those methods applied in at least ten studies were included

The applicability of the methods used in the studies included in this work was assessed in terms of the age of the subjects. As Fig. 6 indicates, there are notable differences among the proposed approaches. While the methods developed by AlQahtani et al. [10], Nolla [12], Willems and Chaillet [14], and Cameriere et al. [23] focused on a very constrained group of patients aged two to 18, approximately, Demirjian et al.’s method [13] could be applied to a wider group of patients of even more than 25 years of age. The methods focused on post-developmental dental features, such as the one proposed by Kvaal et al. [26] or the pulp-to-tooth volumetric ratios, have as their natural field of application those subjects aged 18 to 70, approximately. On the other hand, the automatic methods have been proven to be applicable in a wider age range, covering both the subjects with developing dentitions and the subjects with fully developed teeth.

## Discussion

The oral cavity, and especially the teeth, has been used for decades as they show a high correlation with development patterns. In this regard, great efforts have been made since the late nineteenth century to develop teeth evolution Atlases, with a view not only to the formation of teeth concerning age but also to the sexual dimorphic patterns of that development. The democratisation of radiology led to a number of improvements, one of them being the collection of bigger databases to use in new studies, which in the end increases the statistical significance of the findings. Another major revolution came with the arrival of computers, which brought the possibility of acquiring X-ray images directly in a digital format and, therefore, to speed up the measuring processes.

With the aim of using the structures that are observable in the dental images to estimate the chronological age, different approaches have been followed. Only three studies collected in this work focused on the mandible bone [38, 39, 54], while the rest relied on tooth analysis methods. Among the latter, two groups of works can be noticed, namely those aimed at estimating age in children and young adults [12, 13, 23] and those focused on adults [26, 31, 36]. Moreover, some authors approached the age estimation problem as a classification task instead of a regression problem. In this regard, some studies tried to classify the age of the subjects into two groups, usually with the main purpose of estimating the chances a person has to be older than the legal age. Other works generalised this idea and applied an age classifier with more than two target classes. The usual way to classify the age in both cases was through some modification of a numeric age estimation method [42–44].

As tooth and bone development is reported to depend on factors such as ethnicity or environment, these methods have been evaluated to a greater or lesser extent in different populations around the world. To assess the differences between the different approaches and the population samples used, this work retrieved a corpus of studies applying age estimation methods in dental images published in the last 7 years (from 2016 to 2022). A total of 613 unique studies were obtained, of which 286 were selected after applying the inclusion criteria. The first point to highlight is the great difference in the number of studies that apply each method. For example, methods such as those proposed by Schour and Massler [8] or Blenkin and Taylor [11] were applied in one single work in the evaluated period, which decreases the significance of the results to a large extent.

Regarding the performance obtained by the analysed methods, it has been proved that the eruption-based methods are useful for a very short period (until 15 years of age, approximately), and the only method included in this work, proposed by Haavikko et al. [7], led to a systematic underestimation of the chronological age in every tested population. Similarly, Atlas-based methods assessed have also been applied to very constrained population samples in terms of age, being the London Atlas method [10] the only method evaluated in multiple populations.

Dental staging approaches have proven to be highly relevant in this period, as they accounted for nine of the ten most commonly used methods in this work. This allowed us to compare these methods to each other accurately and confirm the findings reported in previous works, such as the tendency of Nolla [12] and Demirjian et al.’s [13] methods to underestimate and overestimate the age, respectively [77], or the balanced behaviour yielded by the Willems modification of Demirjian’s method [14]. The methods based on Gleiser and Hunt stages [18] achieved slightly worse results than Nolla’s [12] or Demirjian et al.’s [13], while both Moorrees et al.’s [19] and Kohler et al.’s [20] approaches showed a significant underestimation and greater absolute errors. It should also be noted that the method proposed by De Tobel et al. [22], which combines multiple dental staging methods, showed a behaviour with no tendency to underestimation or overestimation, but greater absolute errors than those obtained with the methods it relies on individually. On the other hand, Cameriere’s method [23] based on the measurement of open apices systematically underestimated the age, while the absolute errors were highly inconsistent compared to the previously mentioned methodologies.

Estimating chronological age in adults has proven to be a much more difficult task, as confirmed in the studies retrieved in this work. None of the studies reported absolute errors lower than 2 years, and trends of under- or overestimation were also more pronounced [69, 78]. Regarding the methods using linear or volumetric dental measurements, there is a clear improvement in terms of standard error values when volumetric information is taken into account. It is also noticeable that adult-oriented methods do not report as many performance metrics as those targeted at children, which hinders the assessment of their performance. The most problematic cases are those involving the radiographic visibility methods and the mandible-based methods, where a direct comparison is not possible.

The methods proposed for detecting if someone is younger or older than a predefined threshold (usually the legal age) provided a more suitable environment for comparability purposes, as most of them reported the accuracy, sensitivity, and specificity metrics. The majority of studies yielded reasonably high accuracy, with values over 80 or even 90%, suggesting that these methods could be used either alone or combined with others to produce confident estimations. However, it is worth noting that the results are very dependent on the age cohort used to test the methodology. If subjects are not well-balanced around the threshold, that is, if most subjects are younger or older than the legal age, the performance will be abnormally good, as the classifier will tend to assign the majority class to all subjects [79]. A similar problem can occur if the testing age cohort is too wide, as the further away the subjects are from the age threshold, the easier it is to classify them and, therefore, the better the classification performance.

The inclusion of modern image processing techniques for age estimation led to a set of improvements, being the first one the time and cost saving by means of full or almost-full automation. Moreover, the performance achieved by these methods is clearly higher than that obtained with the manual methodologies. Although in some cases the errors were high due to the exclusion of children from the population sample [51, 53, 55], the overall performance was remarkable, with absolute errors lower than one year for patients younger than 20 [49] or 1.64 years in a huge sample of almost 28,000 images from patients aged zero to 93 years [50]. In this regard, the applicability of these methods is better compared to that of the classic manual approaches, with an almost avoidance of underestimation and overestimation problems and the subjectivity intrinsic to human-guided processes, as well as the possibility to run estimations in a wider age cohort.

The studies that applied automatic methodologies to perform binary age classification or multiple group age classification are not as numerous as those aimed at performing numeric age regression. Moreover, traditional methods in this regard reached very good results, with almost no room for improvement. However, the proposed automated methodologies kept the same high-performance level while providing a series of benefits such as economic and resource-saving [59, 61].

Regarding the specific computational approaches followed by the automatic methods, most studies rely on fully automatic and deep-learning-based solutions, which lead to two key enhancements. As with every end-to-end method, the dataset only needs to be annotated with the expected output, that is, the age, reducing the time of this process and, thus, making it possible to compile bigger datasets of thousands of images. Furthermore, these methods do not rely on specific bone structures designated by an expert, but rather on the image parts that the algorithm considers to be most relevant for that specific task. As there is no need for specific teeth to be present, these methods can work even if some pieces are missing.

Although these automatic methods have been shown to improve the performance and applicability of age estimation methods, their validation needs to be improved. The relative recency of deep learning techniques causes that no automatic method has been tested in populations or acquisition devices other than the original ones, which raises doubts about their generalisation to different scenarios. However, the ease to compile new databases in this regard allows these methods to be easily adapted to different situations through the application of specific domain-adaptation techniques, such as transfer learning or fine-tuning [80].

## Conclusions

In this work, the current application of age estimation methods in recent years has been studied, specifically those methods using radiological dental images. Although classic methods have been thoroughly evaluated in many populations of different ethnicities all over the world, automatic methods based on deep learning techniques led to an improvement not only in terms of performance but also regarding the applicability in a real scenario. This represents a turning point in the field of chronological age estimation, since the speed at which the estimations can be applied can be significantly higher and the subjectivity inherent to the observer analysis can be completely avoided. Future work in this area should involve a deeper assessment of the proposed automatic methodologies, specifically their evaluation in samples of different ethnicities, to improve their generalisation capabilities.

## Data Availability

Not applicable.
